# Metabolomic analysis data of MPP^+^-exposed SH-SY5Y cells using CE-TOFMS

**DOI:** 10.1016/j.dib.2020.106707

**Published:** 2020-12-30

**Authors:** Taku Amo, Yutaka Oji, Shinji Saiki, Nobutaka Hattori

**Affiliations:** a Department of Applied Chemistry, National Defense Academy, 1-10-20 Hashirimizu, Yokosuka 239-8686, Japan; b Department of Neurology, Juntendo University School of Medicine, 2-1-1 Hongo, Bunkyo-ku, Tokyo 113-8421, Japan

**Keywords:** CE-TOFMS, MPP^+^, Metabolome, Mitochondria, Parkinson's disease, SH-SY5Y

## Abstract

1-Methyl-4-phenylpyridinium (MPP^+^)-treated human neuroblastoma SH-SY5Y cells have been generally accepted as a cellular model for Parkinson's disease. This article contains metabolic analysis data of not only cell lysate but also culture supernatants to understand comprehensive metabolic disturbances in this model. Metabolic analysis employed by capillary electrophoresis time-of-flight mass spectrometry (CE-TOFMS). Data obtained by CE-TOFMS were processed to extract peak information including *m/z*, peak area, and migration time. The data provided in this manuscript have been analyzed and discussed in the research article entitled “Metabolomic analysis revealed mitochondrial dysfunction and aberrant choline metabolism in MPP^+^-exposed SH-SY5Y cells” [1].

## Specifications Table

**Table:** 

Subject	Cellular and Molecular Neuroscience
Specific subject area	Metabolomic analysis
Type of data	Table
How data were acquired	Capillary electrophoresis time-of-flight mass spectrometry (CE-TOFMS)
Data format	Raw and analyzed data in the form of .xlsx file.
Parameters for data collection	Differentiated SH-SY5Y cells were treated with 0, 30 or 300 μM MPP^+^. Samples were collected before treatment (0 hour) and after 24 h.
Description of data collection	Culture supernatants and methanol extracts from cells were ultrafiltered through 5 kDa cut-off membrane, and subjected to CE-TOFMS.
Data source location	Tokyo, Japan
Data accessibility	Culture supernatants: Supplementary Table 1
	Cell lysates: Supplementary Table 2
Related research article	T. Amo, Y. Oji, S. Saiki, N. Hattori, Metabolomic analysis revealed mitochondrial dysfunction and aberrant choline metabolism in MPP^+^-exposed SH-SY5Y cells, Biochem. Biophys. Res. Commun. 519 (2019) 540–546.
	https://doi.org/10.1016/j.bbrc.2019.09.031

## Value of the Data

• Both of extracellular and intracellular metabolic profiles provide an overview of the effect of MPP+ exposure to differentiated SH-SY5Y cells, which is an established cellular model for Parkinson's disease (PD).
• The data could be beneficial for researchers working on MPP+-exposed cellular PD models and MPTP (1-methyl-4-phenyl-1,2,3,6-tetrahydropyridine)-exposed animal PD models.
• Metabolic changes elucidated using metabolomic analysis of a cellular PD model may provide our understanding about not only cellular and animal PD models, but also PD pathogenies.
• Especially extracellular metabolic profiles may provide insight into seeking PD biomarker of body fluids like serum/plasma, cerebrospinal fluid or urine.

## 1 Data Description

MPTP is often used to produce animal models for Parkinson's disease (PD) because it produces selective loss of dopaminergic neurons in the substantia nigra and results in irreversible parkinsonism [2]. As a cellular model for PD, human neuroblastoma SH-SY5Y cells treated with MPP^+^, which is a toxic metabolite converted from MPTP, were widely used [3]. This article contains capillary electrophoresis time-of-flight mass spectrometry (CE-TOFMS) metabolomic analysis data obtained from the culture supernatants and the cell lysate of MPP^+^-treated -SH-SY5Y cells [1]. The datasets containing compound name, database ID, m/z, migration time (MT) and relative peak area are provided in Supplementary Table 1A (culture supernatant) and 2A (cell lysate). The relative quantification data of main metabolites are also provided in Supplementary Table 1B (culture supernatant) and 2B (cell lysate).

## 2 Experimental Design, Materials and Methods

### 2.1 Cell culture

Human SY-SY5Y neuroblastoma cells were obtained from American Type Culture Collection (ATCC; Manassas, VA, USA) and cultivated in DMEM – high glucose supplemented with 10% FBS and NEAA. The cells were differentiated with 20 μM *all-trans* retinoic acid (Sigma, St. Louis, MO, USA) for 48 h. After differentiation, 0, 30 or 300 μM 1-methyl-4-phenylpyridinium (MPP^+^; Sigma) was added for 24 h. Samples of culture supernatant and cell lysate were collected before treatment (0 h) and after 24 h, and then subjected to metabolite extraction (each *n* = 3, except *n* = 6 for 0 h-culture supernatant).

### 2.2 Metabolite extraction and metabolomic analysis

The supernatants of culture media were collected and an internal standard (Human Metabolome Technologies (HMT; Yamagata, Japan) was added to them. To remove macromocleules, ultrafiltration was employed using 5 kDa cut-off membrane (UltrafreeMC-PLHCC, HMT) at 9100 × g for 1 h at 4 °C. The filtrate was then provided for metabolomic analysis.

After removing culture supernatant, the cells grown in 90 mm dishes were washed with 10 mL of 5% mannitol solution. Second wash was employed with 2 mL of 5% mannitol solution. The metabolites were extracted with 800 μL of methanol (LC-MS grade) and then 550 μL of water containing internal standard (HMT) was added. 1000 μL of the mixtures were collected and centrifuged at 2300 × g for 5 min at 4 °C. Next, 700 μL of the supernatants were subjected to ultrafiltration to remove macromolecules. The ultrafiltration was employed using 5 kDa cut-off membrane (UltrafreeMC-PLHCC, HMT) at 9100 × g for 2 h at 4 °C. The filtrate was then dried and reconstituted in 25 μL Milli-Q water prior to metabolomic analysis.

Metabolomic analysis was conducted at HMT by CE-TOFMS as described previously [1], [4], [5].

### 2.3 Statistical analysis

The statistical significance between two groups was assessed by Welch's *t*-test. *P* values < 0.05 were considered statistically significant (*P < 0.05; ^⁎⁎^*P* < 0.01; ^⁎⁎⁎^*P* < 0.001).

## CRediT Author Statement

**Taku Amo:** Formal analysis, Writing - Original Draft, Writing - Review & Editing, Visualization. **Yutaka Oji:** Investigation, Resources. **Shinji Saiki:** Conceptualization, Methodology, Writing - Review & Editing, Supervision, Funding acquisition. **Nobutaka Hattori:** Project administration, Funding acquisition.

## Declaration of Competing Interest

The authors declare that they have no known competing financial interests or personal relationships which have, or could be perceived to have, influenced the work reported in this article.

## Appendix Supplementary materials

Image, application 1

Image, application 2
